# Understanding Clinic and Community Member Experiences with Implementation of Evidence-Based Strategies for HPV Vaccination in Safety-Net Primary Care Settings

**DOI:** 10.1007/s11121-023-01568-4

**Published:** 2023-06-27

**Authors:** Jennifer Tsui, Michelle Shin, Kylie Sloan, Bibiana Martinez, Lawrence A. Palinkas, Lourdes Baezconde-Garbanati, Joel C. Cantor, Shawna V. Hudson, Benjamin F. Crabtree

**Affiliations:** 1 University of Southern California, Los Angeles, CA USA; 2 University of Washington, Seattle, WA USA; 3 Rutgers the State University of New Jersey, New Brunswick, NJ USA

**Keywords:** Implementation of evidence-based strategies, HPV vaccination, Multilevel assessment, Primary care, Practice change model

## Abstract

HPV vaccination rates remain below target levels among adolescents in the United States, which is particularly concerning in safety-net populations with persistent disparities in HPV-associated cancer burden. Perspectives on evidence-based strategies (EBS) for HPV vaccination among key implementation participants, internal and external to clinics, can provide a better understanding of why these disparities persist. We conducted *virtual interviews and focus groups*, guided by the Practice Change Model, with clinic members (providers, clinic leaders, and clinic staff) and community members (advocates, parents, policy-level, and payers) in Los Angeles and New Jersey to understand common and divergent perspectives on and experiences with HPV vaccination in safety-net primary care settings. Fifty-eight interviews and seven focus groups were conducted (*n* = 65 total). Clinic members (clinic leaders *n* = 7, providers *n* = 12, and clinic staff *n* = 6) revealed conflicting HPV vaccine messaging, lack of shared motivation to reduce missed opportunities and improve workflows, and non-operability between clinic electronic health records and state immunization registries created barriers for implementing effective strategies. Community members (advocates *n* = 8, policy *n* = 11, payers *n* = 8, and parents *n* = 13) described lack of HPV vaccine prioritization among payers, a reliance on advocates to lead national agenda setting and facilitate local implementation, and opportunities to support and engage schools in HPV vaccine messaging and adolescents in HPV vaccine decision-making. Participants indicated the COVID-19 pandemic complicated prioritization of HPV vaccination but also created opportunities for change. These findings highlight design and selection criteria for identifying and implementing EBS (changing the intervention itself, or practice-level resources versus external motivators) that bring internal and external clinic partners together for targeted approaches that account for local needs in improving HPV vaccine uptake within safety-net settings.

## Introduction

Despite evidence that HPV vaccination can prevent infection of high-risk HPV types associated with several cancer types (Dehlendorff et al., 2021; Xu et al., 2019), HPV vaccination among adolescents continues to be suboptimal in the United States (US). National data suggest vaccine initiation rates have steadily increased in recent years (Pingali et al., 2021), with 75% of adolescents (13–17 years) having received at least 1 dose and 59% completing the series in 2020. More granular data by race and ethnicity, socioeconomic status, acculturation, and geography, however, indicate adolescents from medically underserved communities have lower rates of HPV vaccination (Btoush et al., 2015; Glenn et al., 2015; Kim & LeClaire, 2019; Wilson et al., 2015). This is particularly concerning in underserved populations where disparities in higher cervical cancer incidence and mortality remain (Dehlendorff et al., 2021; Meites et al., 2016).

An increasing number of evidence-based strategies (EBS) for improving HPV vaccination have emerged over the past decade (Chao et al., 2010; Dempsey et al., 2018; Niccolai & Hansen, 2015; Perkins et al., 2015; Smulian et al., 2016; Vadaparampil et al., 2011). These provider and health system interventions are not only effective in improving uptake among adolescents but also often play stronger roles than efforts addressing parental knowledge or beliefs alone (Fu et al., 2014). In particular, multi-component intervention strategies (Bastani et al., 2022; Kepka et al., 2021; Niccolai & Hansen, 2015; Perkins et al., 2021; Walling et al., 2016) that target several aspects of the healthcare setting, including missed clinical opportunities (Perkins et al., 2014; Vadaparampil et al., 2011), physician recommendation quality (Dempsey et al., 2018; Gilkey et al., 2016), and use of reminder systems (Rand et al., 2017) have the strongest impact on increasing HPV vaccination among target age groups. However, barriers to implementation of HPV vaccination strategies related to local context and population fit are not well understood, including how HPV vaccination is prioritized compare to other adolescent health topics and whether increasing vaccine hesitancy across communities interact with the feasibility and importance of specific EBS. Vaccine hesitancy, defined as a “delay in acceptance or refusal of vaccines despite availability of vaccine services” is an increasing public health challenge (Hickler et al., 2015; Strategic Advisory Group of Experts on Immunization, 2014). Prior work by our team in both Los Angeles and New Jersey has identified parental HPV vaccine hesitancy (Tsui et al., 2022) and providers’ needs to address parental concerns (Tsui et al., 2021) as additional factors that require further exploration in optimizing clinic-based strategies for HPV vaccine promotion. As EBS for increasing HPV vaccination continue to emerge in healthcare, it is critical to understand both clinic and community level contexts (e.g., internal clinic resources, external influences, and barriers to change) required to successfully implement and sustain these strategies in safety-net settings serving diverse communities. Thus, a comprehensive understanding of perspectives of clinic and community members on and experiences with EBS for HPV vaccination in safety-net primary care settings are needed to inform implementation efforts and address equity in HPV vaccine uptake.

Achieving target population-level adolescent HPV vaccination rates of 80% (Centers for Disease Control and Prevention, 2020) increasingly relies on understanding the influence of multiple actors and organizations both internal and external to clinic settings (Anderson et al., 2005; Cohen et al., 2004; Crabtree et al., 2011). Prior studies focusing on understanding implementation of clinic-level strategies for HPV vaccine improvement have primarily examined provider experiences (Escoffery et al., 2019; Garbutt et al., 2018; Selove et al., 2017). Comprehensive assessment of the broader implementation context, however, requires gaining perspectives that also represent organizational and community-level influences. Perspectives of policymakers, payers, advocates, and parents can further elucidate specific contextual barriers to implementation of HPV vaccination strategies within clinics, such as resource constraints in expanding vaccine access and community-specific information needs to guide decision-making among parents. Since little is known about the perspectives of these different interest groups, especially differences across geographic areas with diverse populations, we conducted a qualitative study (interviews and focus groups) to more deeply understand clinic and community members’ experiences with EBS for HPV vaccination in safety-net healthcare settings, with the goal of informing adoption and adaptation needs across clinics and contexts.

## Methods

### Study Overview and Conceptual Framework

We leveraged the unique opportunity of established relationships between our multidisciplinary research team and HPV vaccine-relevant clinic and community partners in both Los Angeles and New Jersey to focus on two geographic areas with large racial/ethnic and socioeconomic diversity. These areas have suboptimal HPV vaccination rates coupled with a history of advocacy and motivation to improve vaccine uptake, and a significant proportion of vulnerable communities are served by safety-net settings. We conducted *virtual one-on-one semi-structured depth interviews with participants* internal and external to safety-net clinic settings who influence HPV vaccination strategies and uptake in the greater Los Angeles region (LA) and New Jersey (NJ), from six groups: providers (MD and NP), clinic leaders, clinic staff (registered nurse, medical assistant, and navigators/health educators), advocates, payers (health plan executives and health plan population–health directors), and policy-level representatives. We also conducted a combination of *virtual focus groups and virtual one-on-one semi-structured depth interviews with parents of adolescents.*

**Fig. 1:**
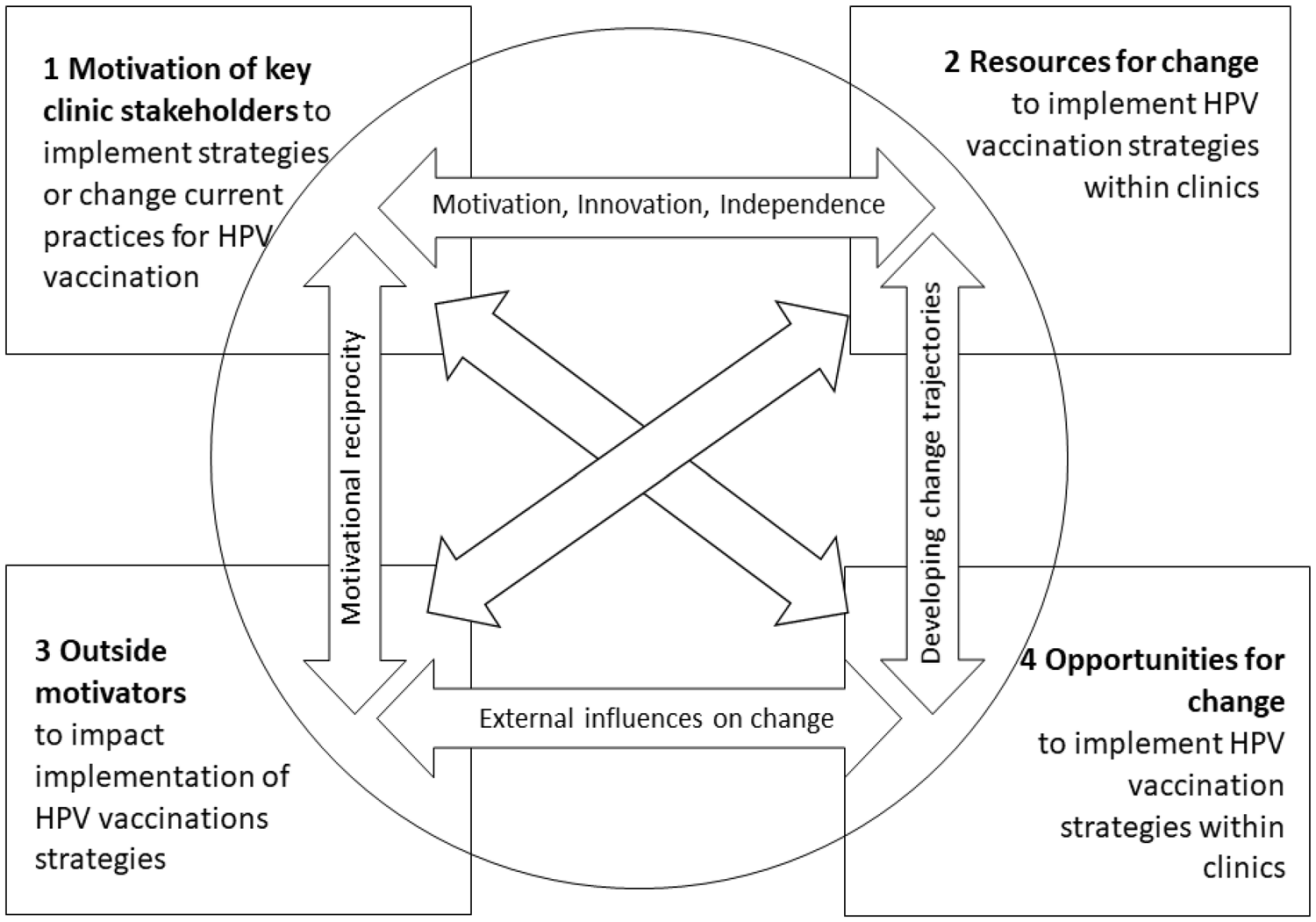
Adapted practice change model as a guiding framework for understanding implementation of evidence-based strategies for HPV vaccination within safety-net clinics

We used the practice change model (PCM) as a theory-based framework to guide interviews to examine internal and external practice-based factors that impact adoption of EBS and the interrelationships among these factors (Cohen et al., 2004). PCM domains included the following: (1) motivation to implement EBS for HPV vaccination in clinic settings, (2) existing resources to promote EBS for HPV vaccination in clinic settings, (3) clinics’ motivators for implementing EBS or how participants serve as outside motivators in clinic settings, and (4) noted opportunities for change within clinics to implement or promote EBS and interrelationships across domains (1) through (4) (arrows) **(**Fig. 1).

### Sampling Strategy and Recruitment

We used purposive, snowball sampling techniques to identify and recruit participants across each geographic area (LA, NJ), with a target of 50–70 total participants, consisting of six to ten members per group. We identified clinic and community members, at least 18 years of age, from regional immunization and HPV vaccine coalitions as well as networks of investigators, consultants, and advisory group members on the research team. We sought individuals based on their involvement with HPV vaccination or adolescent health as well as experience or reputations of being HPV vaccine champions, presuming they would best be able to speak about experiences with HPV vaccination. We aimed to maximize diversity in organization type (e.g., large vs. small clinics), populations (e.g., racial/ethnic subgroups), and geographic areas (LA and NJ). We recruited an initial wave of clinic members (internal setting), including providers and clinic leaders, followed by community members (external setting), including policy-level representatives, payers, advocates, and parents. Clinic staff were recruited last, based on referral from providers and clinic leaders, to accommodate COVID-19 pandemic disruptions and priorities in clinic settings. After completing the first wave of interviews for each group, we reviewed interview and focus group content, emerging themes, and assessed for thematic saturation to inform additional recruitment. Thus, *participants for each group were contacted and scheduled to be interviewed virtually* until theoretical saturation was reached (i.e., no new information was obtained from participants) (Crabtree, 1999). Previous studies relying upon this methodology have typically found that information becomes repetitive, and little new information is gained after analyses of data collected from as few as 12 respondents (Guest et al., 2006). Participants in the first wave of interviews also served as referral sources for a second wave of confirming and disconfirming interviews (Cohen & Crabtree, 2008; Dicicco-Bloom & Crabtree, 2006). The research team intended to conduct focus groups for parents in both LA and NJ; however, one-on-one interviews were completed in NJ due to participant availability during the pandemic. We aimed to conduct two focus groups per language, one with parents or guardians with at least one HPV-unvaccinated adolescent child (ages 9–17) and another with parents of at least one HPV-vaccinated child.

### Human Subjects

Study investigators and project team members made initial contact with potential participants via email to arrange virtual interviews and parent focus groups. All potential participants were contacted up to three times before classifying them as non-respondents. *All interviews and parent focus groups were conducted virtually, *via* Zoom between December 2020 and January 2022.* Each participant received a $50 gift card incentive upon participation completion. Interviews and focus groups were approximately 30–60 min. This study was approved under exempt protocol status through the University of Southern California and Rutgers University Institutional Review Boards.

### Researcher Characteristics and Reflexivity

As Crabtree and Miller note, “systematic reflexivity needs to happen with each iteration and at each location throughout the research process,” which we accomplished by regularly meeting as a team and sharing our preconceptions and how they changed over time (Crabtree, 2023). Our multidisciplinary, qualitative data team encompassed junior and senior investigators with training in nursing science, medical sociology, behavioral health, social work, medical anthropology, and cancer health services research. Team members had varying degrees of comfort, personal support, and positionality in relation to vaccination and experiences with clinic and community settings, which were reflected upon throughout group analyses and data interpretation.

### Data Collection

Interviews followed guides, which were constructed based on the PCM domains and relationships across domains described above (Cohen et al., 2004), our prior work (Anuforo et al., 2022; Tsui et al., 2021, 2022), and with input from the research team and consultants. Interview guide questions included topics related to past experiences with providing and recommending HPV vaccines, strategies currently used within the participant’s organization to improve HPV vaccination rates, specific changes participant would like to see in HPV vaccination processes, interactions with external organizations that influence HPV vaccine improvement, and practice change requirements needed to implement new strategies for HPV vaccination. Interview guides were first constructed for providers and clinic leaders and then adapted for subsequent groups. Guides were piloted internally with research team members and community collaborators for clarity and estimated duration.

*Parent focus groups were conducted virtually in LA* and used a guide adapted from parent interview guides to prompt descriptions of experiences with vaccination for adolescent children, HPV vaccine knowledge and attitudes, barriers to vaccination, and exposure to clinic-based EBS (e.g., patient reminders). The focus group guide was translated into Spanish and Chinese (Mandarin) and reviewed by multilingual/cultural community members for appropriateness. The focus groups were conducted virtually by a facilitator and a notetaker who were fluent in the target language.

### Data Analysis

All interviews and focus groups were audio-recorded, transcribed verbatim by an external transcription service, and then de-identified and checked for accuracy by interviewers. Interview transcripts were analyzed through several rounds of an immersion/crystallization approach (Crabtree, 2023) by investigators and research team members (described in reflexivity section above). Transcripts were distributed among team members and reviewed thoroughly to develop a broad understanding of content related to the project’s aims and to identify emerging themes within a particular group. We then used a summary document or “memo” for each participant group (i.e., clinic leaders, providers, advocates, and payers) to document initial topics and emerging themes structured around the PCM domains (Fig. 1) and inform the boundaries of specific codes. Upon completion of the memo for each participant group, the team met to discuss emerging themes and assessed for within group saturation (e.g., number of providers who raised an issue). Then, the team held subsequent crystallization meetings to compare memos across participant groups and assess saturation between groups (e.g., number of providers, advocates, payers, and parents who raised an issue). A priori codes based on the interview guide and emerging topics/codes from the memos and immersion process were discussed during the crystallization meetings to produce a single codebook for all transcripts. Transcripts were then independently coded to condense the data into analyzable units using Atlas.ti version 9.

The analysis team coded two transcripts together to refine code definitions and calibrate code usage. When team members reached group consensus on the same codes for highlighted segments of text, the transcripts were independently coded by two members of the research team. The qualitative data manager reviewed the coded transcripts to identify discrepancies in code usage. Disagreements in code assignments were resolved through discussions and meetings where team members re-read portions of text to refine code definitions or come to consensus. The final codebook, constructed through team consensus, consisted of a list of topics, issues, accounts of behaviors, and opinion experiences and perceptions related to HPV vaccination and EBS to improve vaccination. Thereafter, we divided the remaining transcripts among the team members to code individually, meeting bi-weekly to discuss coding questions and review portions of ambiguous text. *After all, data had been summarized and coded, team members individually immersed themselves in the code output as well as the summaries to identify patterns across participant groups,* using the process of constant comparison to construct clusters of codes, and then met throughout multiple meetings to collectively crystalize the most salient themes.

## Results

### Participants

A total of 58 qualitative interviews were conducted across groups of providers, clinic leaders, clinic staff, advocates, policy-level, payers, and parents in LA (*n* = 37) and NJ (*n* = 28). Table 1 shows the organizational and role characteristics of participants, the number of participants per group, and the sources of recruitment and demographic information of parent participants. Overall, providers practiced in diverse specialties (e.g., primary care, obstetrics gynecology, and pediatrics in both acute and outpatient clinics) within safety-net settings. Many clinic leaders were also physicians who held clinical and administrative leadership roles within practices. Clinic staff included nurse managers, community health workers, vaccine coordinators, and medical assistants, and all were from federally qualified health centers (FQHCs). The advocates worked in both governmental/non-governmental organizations (e.g., American Cancer Society). Policy-level representatives, including policymakers and policy implementers, included individuals in local- and state-level legislator and representatives’ offices or local and state health departments or school district affiliated programs. Payers were mostly physicians working in insurance organizations and local public insurance programs. Parent interviews were conducted in the focus group format in LA (*n* = 7 focus groups with 38 parents total) and as one-on-one semi-structured interviews in NJ (*n* = 6). In LA, most of the parent participants were female caregivers/mothers of multiple adolescents ages 9–17, whereas half of the NJ parents were male caregivers/fathers. LA focus groups were conducted in English (1 vaccinated and 1 unvaccinated), Spanish (2 vaccinated and 1 unvaccinated), and Mandarin (1 vaccinated and 1 unvaccinated).

**Table 1:** Summary of interview and focus group participants in LA and NJ (*n* = 65)

**Participant group**		**Region**	***N***	Study ID^*^	**Organizations represented**
Clinic (internal setting) members	Providers	LA	5	LA03, LA04, LA14, LA17, LA22	County System-Pediatric Clinic, Academic Health System-FQHC, FQHC
		NJ	7	NJ01, NJ02, NJ03, NJ04, NJ05, NJ21, NJ28	Private Practices, Hospital System-Pediatrics, Academic Health System-Pediatrics, Academic Health System FQHC, FQHC
	Clinic leaders	LA	3	LA02, LA05, LA13	FQHC, Hospital Run Pediatric Vaccine Mobile Program, Children’s Hospital-FQHC Clinic
		NJ	4	NJ13, NJ17, NJ18, NJ22	Hospital System-Pediatrics, Hospital System Administration
	Clinic staff	LA	5	LA18, LA19, LA28, LA29, LA30	FQHC QI Leaders, FQHC Nurse Manager, FQHC Medical Assistant, FQHC Community Health Worker
		NJ	1	NJ19	FQHC Immunization Coordinator/Medical Assistant
Community (external setting) members	Advocates	LA	4	LA06, LA07, LA08, LA09	Non-profit Foundation Regional Manager, Statewide Cancer Control Leader, School-Based Health Non-Profit Organization Program Officer
		NJ	4	NJ06, NJ07, NJ08, NJ09	Non-profit Foundation Regional Manager, Statewide Immunization Coalition Leader, Immunization Advocacy Organization Specialist, Statewide Maternal/Child Health Partnership Officer
	Policy	LA	8	LA01, LA10, LA11, LA12, LA16, LA21, LA23, LA25	State Immunization Registry Educator, Statewide Cancer Control Coalition Leader, County State Immunization Program Medical Officer, Leader of California School-Based Health Non-Profit Organization, Leader of School District Wellness Center, Health Deputy for County Supervisor, Chief of Staff for City Councilmember
		NJ	3	NJ10, NJ11, NJ12	Chief of staff for state legislator. State Department of Health Representatives
	Payers	LA	5	LA15, LA20, LA24, LA26, LA27	Medi-Cal Managed Care Organization, County Medi-Cal Plan, Consulting Firm for Publicly Funded Healthcare
		NJ	3	NJ14, NJ15, NJ16	Medicaid Managed Care Organization, Commercial Health Plan, State Medicaid Program
	Parents	LA	7	7 FGs (*n* = 38 parents total)	Parents of unvaccinated children (3 FGs) and vaccinated/initiated children (4 FGs): • English: 1 unvaccinated (*n* = 4), 1 vaccinated (*n* = 3) • Spanish: 1 unvaccinated (*n* = 7), 2 vaccinated (*n* = 11) • Mandarin: 1 unvaccinated (*n* = 4), 1 vaccinated (*n* = 5)
		NJ	6	NJ20, NJ23, NJ24, NJ25, NJ26, NJ27	Parents (racial/ethnic minority, immigrant) with children that were fully vaccinated, initiated, and unvaccinated

^*^Only parents in LA participated in focus groups, all other participants conducted one-on-one interviews

Ten themes emerged from our analysis, which are organized around the PCM and their interrelationships. These themes are depicted in the following three broad categories: internal motivators and barriers to implementation of HPV vaccination strategies within clinics (themes 1–3), focusing on clinic member (providers, clinic leaders, and clinic staff) perspectives as they relate to PCM Domains 1, 2, and 4; external motivators and barriers to implementation of HPV vaccination strategies within clinics (themes 4–7), from perspectives of community members (advocates, policy-level, payers, and parents) as they relate to PCM Domains 2, 3, 4; and interdependencies across groups and the impact on implementation of strategies (themes 8–10) as they relate to PCM arrows (Fig. 1).

### Internal Motivators and Barriers to Implementation of HPV Vaccination Strategies Within Clinics

#### Theme 1: Conflicting Messaging of HPV Vaccinations Complicates Strategies to Improve Uptake

Participants mentioned that two primary messages for benefits of HPV vaccination, the focus on cancer prevention versus the focus on preventing a sexually transmitted disease, were used inconsistently across groups, limiting a coherent overall message about the vaccine. The cancer prevention message had been widely adopted by internal clinic members and actively supported by advocates in recent years, in part to destigmatize the vaccine by removing the STI focus. Providers explained that they believed this shift to cancer prevention messaging helps to increase vaccine uptake because they “*find it to be a more receptive message for parents than saying, ‘Oh, this will prevent your daughter or son from getting an [STI].’ They look at me like, ‘Uh, they shouldn’t be doing anything to get an [STI]’”* (LA17, Provider). However, clinic members who serve in sexual education and reproductive health settings (e.g., school-based settings, obstetric gynecology or other specialty care clinics, or for LGBTQ+ populations) discussed that it was difficult to promote or administer the HPV vaccine in their context with the cancer prevention messaging. In LA, adolescents have the ability to receive the HPV vaccine for themselves through minor consent. Adolescent clinics funded through reproductive health initiatives thus used STI messaging about the vaccine because *“in California, [for adolescents] 12 years and older, you can actually assent for the HPV vaccination because it has to do with your reproductive health” (LA14, provider).*

Furthermore, while some providers and clinic leaders endorsed starting HPV vaccination at age 9, instead of age 11, others did not see the benefit or struggled to get buy-in from clinic staff in their practice. One provider stated that starting HPV vaccination at age 9 interfered with the bundling strategy:

*In our clinic, we do it at the 11-year visit. We did talk about going down to the 9-year visit. Unfortunately, we’ve not been able to convince all our clinic staff. Clearly, we need buy-in from our clinic nurses and staff…I think because with the 11-year, they have the other vaccines, so I think for the nurses, it’s just easier to give everything at the same time. (NJ05, provider).*

The conflicting perception and endorsement of starting HPV vaccination at age 9 seemed to hinder consistent adoption of this strategy across the clinics. Although advocates and the majority of providers and clinic leaders had shifted the focus of HPV vaccination from STI prevention to cancer prevention, HPV viewed as a STI was still a prominent barrier that limited consistent messaging by clinic staff and contributed to parental hesitancy.

#### Theme 2: Motivation to Address Missed Opportunities for HPV vaccination During Clinic Encounters but Some Strategies Interfere with Clinic Workflows

There was strong emphasis among providers, clinic leaders, and advocates in both regions to focus on reducing missed opportunities for HPV vaccination. Multiple clinic leaders mentioned that they perceived the most effective strategy in improving HPV vaccination to be minimizing missed opportunities:

*I think the biggest problems tend to be missed opportunities and people waiting for well-visits in order to update preventive measures. So, rather than [just] seeing an 11-year-old in the office for a cold or a sore throat, saying, ‘You need a flu shot and you should get an HPV vaccine, too.’ (NJ01, provider)*

To reduce missed opportunities, clinics described implementing provider reminders and standing orders. Standing orders allowed trained healthcare professionals other than a provider to administer vaccinations, according to a protocol per state law (Stewart et al., 2016). Some practices also implemented routine chart prep. One clinic leader explained they are *“doing missed opportunity reports by provider. And that has a huge impact”* but that it is “*very challenging to do this [as they] have to go into each chart to double-check because we don’t want to give the data if it’s not valid”* (LA02, clinic leader). Competing priorities within clinics, such as training residents in immunization guidelines, were seen as conflicting with such strategies, leading some clinics not to implement them:

*The standing order was actually something that we thought about doing and then we decided not to because our particular site is a teaching site. And, we want the residents to know their immunization schedule and not just rely on standing orders to let them know what’s due. (LA13, clinic leader)*

#### Theme 3: Lack of Interoperable Electronic Health Records (EHR) and State Immunization Registry Information Creates Data Fragmentation and Limited Monitoring and Feedback Opportunities

Many clinic members highlighted the use of EHR and immunization registry data to monitor and provide feedback on HPV vaccinations rates. Some clinic staff mentioned this is routinely used *“at every pediatric visit”* and that they* “check CAIR [California Immunization Registry] for their history of vaccinations across any practice they've ever visited”* (LA19, clinic staff). However, one FQHC highlighted barriers to providing real-time data and its interface with CAIR, noting that bi-directional interfacing with the registry data was resource consuming:

*We do have the one-directional interface from NextGen to CAIR, but to implement the bi-directional interface, it’s actually a [time-consuming] process that we don’t complete on every single patient. (LA02, clinic leader)*

Another county-system affiliated provider also indicated interoperability issues between their clinic EHR and CAIR “is just bad, very inaccurate” (LA03, provider). Providers also mentioned that altering staff workflow to monitor HPV vaccine rates using EHR was time and resource consuming. This limited the ability of clinics to accurately track vaccine uptake:

*It’s a little bit easier in our inpatient setting [to run reports on vaccination rates], but in the clinic, we don’t have a designated person to run those [reports], but I think it’s important enough that we should. (NJ05, provider)*

Many clinic members also noted they wanted to update the provider prompts to reflect the latest evidence by starting the recommendation at age 9, but making changes in the EHR was difficult because *“the language of the immunization registry says something to the effect of ‘could be given,’ but it doesn’t say it’s due until age 11” and so they have “been working to change that language, but that’s not something that we can independently do”* (LA03, provider*).* Despite clinic members having motivation to use clinic records and state immunization registry data to monitor rates and identify where providers and patients needed to be prompted, data interoperability and the staffing and workflow needs for data abstraction limited the implementation of EBS.

### External Motivators and Barriers to Implementation of HPV Vaccination Strategies Within Clinics

#### Theme 4: Payers Lack Motivation for Change in HPV Vaccination Performance Within Clinics Aside from Acknowledging the Importance of HPV Vaccination and Relevance to Health Equity

Providers and clinic leaders consistently recognized Healthcare Effectiveness Data and Information Set (HEDIS) measures from the National Committee for Quality Assurance (NCQA) as important motivators to monitoring adolescent vaccination rates because one of their *“quality measures is the adolescent combination vaccine, which is a HEDIS measure”* and so part of their *“focus is to improve immunization rates in adolescents and to meet our quality goals”* (LA13, clinic leader). Payers, however, had varying knowledge about specific adolescent immunization measures (e.g., eligibility age and number of doses). The current adolescent immunization metric, referred to as Combination 2 (Combo 2), combines all adolescent recommended vaccines (e.g., one dose meningococcal (MCV4); one dose tetanus, diphtheria, and acellular pertussis (Tdap); and two dose HPV by age 13), into one quality measure (NCQA, 2022). Some payers recognized that this metric makes it difficult to motivate improvements in HPV vaccination specifically. For example, while insurance plans may choose to use pay for performance incentives for adolescent immunizations, these initiatives target adolescent vaccinations generally, rather than the HPV vaccine alone. Payers acknowledged overall adolescent immunization rates are low because of low HPV vaccination rates, but indicated limited motivation to address this within their incentive structure:

*We’re trying to use the financial reward and providing them with the strategies and the resources to overcome those low vaccination rates...If you look at the 75th percentile, in 2021 it was 35%. That’s still a dismal number. The 90th percentile only 42[%], so we’re less than 50% that have gotten all those vaccinations. The HPV [vaccine] is the barrier. The HPV rates are always lower than the meningococcal and the tetanus. (NJ14, payer)*

While some participants noted there is an opportunity for change to improve HPV vaccination through more targeted HPV vaccine incentives and focus, payers acknowledged that adolescent immunizations, and HPV vaccination specifically, are not at the top of priorities for payers and other safety-net clinic funders because *“if CMS or any of the other regulators or accreditation bodies were to say, ‘HPV vaccination rates is the most important thing and will account for 30, 40, 50 percent of your overall performance rating’ – that would have a huge external influence”* (LA20, payer).

Overall, payers saw themselves as having minimal roles in terms of HPV vaccine promotion aside from vaccine reimbursement and existing quality metrics. Some participants exhibited awareness of the larger role payers have in influencing focus on increasing HPV vaccination in clinic settings. A consistent lack of motivation for change emerged as a result of varied awareness of resources and opportunities for improving HPV vaccination, most often due to competing priorities pulling focus away from adolescent immunizations (e.g., COVID-19 vaccines).

#### Theme 5: Advocates Lead Agenda Setting at the National Level and Implementation of Strategies at the Local Level

Advocates and clinic members noted the important role of state and local advocacy organizations, including the American Cancer Society (ACS) and other non-profit organizations, in convening coalition efforts, serving as liaisons with state and local health departments, and working directly within clinic settings to promote HPV vaccine improvement initiatives:

*I think it also helps just having that ACS branding. Certainly, these groups networking, meeting experts that can deliver some of our provider trainings. We’re all working together, and working together, you’re able to accomplish a lot more than working independently so we definitely see the importance of that. (LA06, advocate)*

Specifically, advocates acknowledged their lead role in disseminating information on effective strategies as well as working with local clinics to fully implement new initiatives:

*We do have toolkits we’ve developed at the ACS so it's all evidence-based. We have an HPV roundtable nationally, so we have resources that we will provide to [them] and present to our primary care and pediatric providers, and it gives them a good overview of what they could be doing. And it's not just a one-and-done meeting. It’s kind of like a series of meetings when we work with them. (NJ07, advocate)*

Beyond practice support and facilitation, local advocacy organizations also encouraged practice change by directly funding new clinic initiatives on HPV vaccination within safety-net settings, where resources are limited. As one advocate shared that FQHCs “*appreciate the funding, and they’re able to really focus their energy” (NJ07, advocate).* Advocates indicated feeling caught between what they believed was most needed, based on national agenda, and what was most actionable or feasible in the local clinic context for HPV vaccination initiative, leading this group to fill the role as both internal and external motivators depending on the needs of the practice setting:

*Part of the funding is that they have to put intervention teams together, right, that they need to have that implementation team. That is not always easy because the team needs to have ‘a population health person’ or someone who oversees their data. Not everyone is familiar at the practice. (NJ07, advocate)*

#### Theme 6: Advocacy, School, and Policy Presence in HPV Vaccination Differ Across Regions and Indicate the Need for Implementation Champions and Attention to Local Context

External community members from the advocacy and policy groups frequently discussed relationships with communities and local (state and county) health departments as important partners in championing HPV vaccination efforts within local contexts and geographic areas. While advocates and policy-level representatives in LA described a strong state cancer control plan and existing state and county HPV vaccine coalitions, advocates in NJ indicated room for improvement in strengthening the focus of local immunization coalitions and state-wide cancer prevention goals:

*I think the [HPV vaccine] roundtables are always good. Any coalitions or roundtables or any coalition-building, it’s always good to meet like-minded advocacy groups. And so, that’s typically how we’ve met them. NJ’s a little different because we don’t have an active comp[prehensive]-cancer taskforce or work group, that typically you would see in other states. (NJ07, advocate)*

Additionally, policy-level representatives discussed how resource allocation decisions are based on local competing priorities and *“squeaky wheels” (LA21, policy).* They noted the important role of advocates impacting policymakers’ decision-making through raising awareness and directing their attention to make HPV vaccination a priority. For example, policy-level participants in LA emphasized the importance of state and county immunization coalitions, led in partnership with public health departments, for increasing awareness in local communities, including engagement through local ethnic and in-language media:

*Coming from a heavily Latinx district, it’s making sure that there is that lens that our health departments are using to make sure they are engaging with ethnic media, hyper-local media, media people are consuming that is outside the mainstream. (LA21, policy)*

Policy-level representatives also described the impact of state policies on minor consent and religious exemptions for vaccines that impact as well as complicates adolescent access to HPV vaccination. In LA, minor consent laws across the state of California allow schools to provide the HPV vaccine to adolescents age 12 and older. However, the Vaccines for Children Program (VFC) requires parental consent for data reporting. This leaves local clinics the ability to provide the HPV vaccine directly to adolescents without parental consent, but through non-traditional primary care channels:

*HPV ends up being in this kind of a strange place and where it kind of is part of the sexual health services that should be covered under the minor consent [laws in California] and then it could also be considered to be covered as a regular immunization which would need parental consent. (LA08, advocate)*

In contrast, NJ currently does not have minor consent laws, prohibiting HPV vaccines from being administered to adolescents without parental consent, thus requiring a different approach and set of avenues for implementation of HPV vaccination strategies suited to local context and policies.

#### Theme 7: Community Members (Advocates, Policy, and Parents) Indicated an Opportunity to Engage Adolescents as Vaccine Decision-Makers

Advocacy and policy-level representatives in both regions emphasized the need to engage with schools, school clinics, and school leaders to empower adolescents, increase access to vaccination through school-based clinics, and provide education to parents. Local advocates in LA, for example, were optimistic about efforts to engage schools beyond vaccination mandates:

*I'm all about just going right into them – right into the high schools, right into the middle schools. ‘Cause if they are given the tools, or helped to acquire the tools independently that speak to these issues, they have credibility that other people will never have ‘cause this directly affects their health. (LA12, policy)*

Parents also specifically mentioned schools as a trusted source of information and motivation to engage their adolescent child in the decision-making process because “*if the school starts talking to the kids, the kids might go home and ask the parents, ‘They told us about this.’ And then the parent might start looking into it” (LA Focus Group, Parent of unvaccinated child).*

Some participants, however, highlighted multiple policy and infrastructure barriers that still need to be addressed to achieve delivery of HPV vaccines and HPV vaccine education through school-based settings*,* including that *“bundling for adolescent immunizations, especially through school clinics is more logistically difficult for stocking. The Family PACT Program in California does not cover HPV vaccine. So, that’s a huge barrier, because the clinics cannot implement minors’ right to consent services without having to eat the cost of the vaccine” (LA16, policy).*

### Interdependencies Across Groups and Impact on Motivation and Resources for Practice Change in Implementation of HPV Vaccination Strategies

#### Theme 8: Motivation for HPV Vaccine Improvement Within Clinics Varied Across Regions due to Available Resources for Change in the Broader Regional Context

Notable regional similarities and differences were observed across internal clinic member groups that signaled varying local context, motivation, and infrastructure for change in HPV vaccination within safety-net settings. Overall, providers in LA practiced in larger, county health system-affiliated institutions (e.g., county pediatric clinic), whereas those in NJ held private practices or worked in clinics affiliated with academic health systems. This may have contributed to perceived resources for change at the clinic or health system level. Clinic leaders in both regions emphasized the importance of minimizing missed opportunities to improve HPV vaccination rates (Theme 2), and many were familiar with HEDIS and other adolescent immunization measures (Theme 4) but only some providers and clinic leaders in LA (LA02, LA04) mentioned opportunities for change in metrics and workflow within clinics.

Providers and clinic leaders in both regions supported HPV vaccine improvement and several clinic members were HPV vaccine champions within the local region/state context (e.g., members of local HPV vaccine coalitions and roundtable initiatives). However, specific motivation for change depended on perceived opportunities with external resources and infrastructure. For example, providers in LA referred to trainings from the state HPV Vaccine Roundtable and in both regions noted funding from local advocacy foundations (e.g., ACS) (Theme 5).

**Fig. 2:**
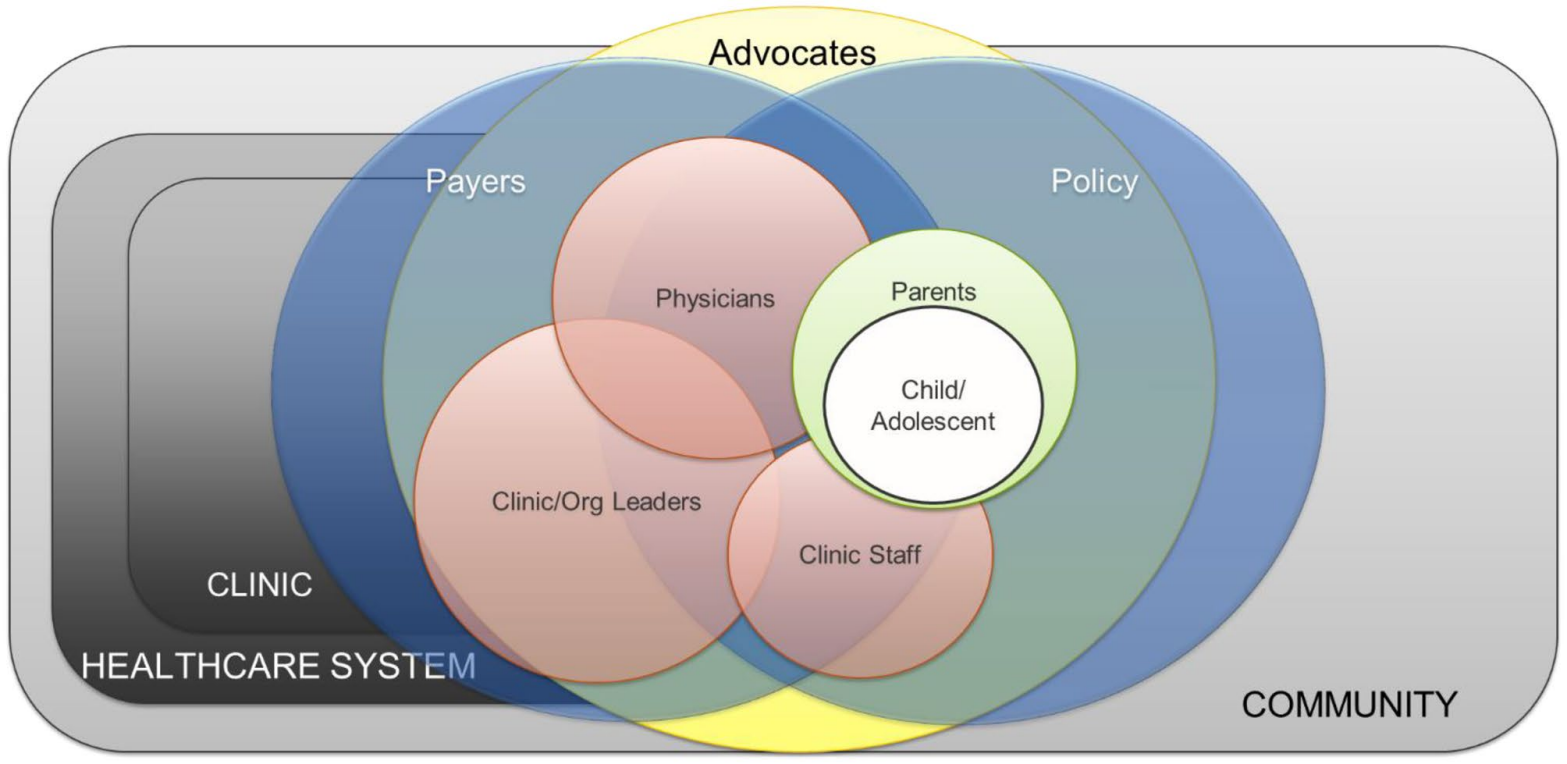
Overlapping interactions and interdependencies across clinic and community groups for HPV vaccination within safety-net settings

Notably, there were perceptions of unclear/overlapping roles of clinic members related to conflicting messaging (Theme 1), particularly among providers across specialties in pediatrics/adolescents/family medicine vs. reproductive health/infectious disease and those who serve children with special needs. Although all providers and clinic leaders supported HPV vaccination, those in specialty settings or non-traditional primary care settings faced barriers to vaccine supply, reimbursement (school-based clinics) and perceived responsibility. Figure 2.

#### Theme 9: Lack of Specific Focus on HPV Vaccination from External Community Members Limits Motivation Among Internal Clinic Members and Opportunities for Change Within Clinics

Regional differences emerged from external community members (advocates, policy, and payers) and indicated an increased need to address hyper-local HPV vaccination context. At the community level, policy-level representatives in both LA and NJ mentioned past and current legislative actions that impact HPV vaccination at the state level (e.g., school mandates and religious exemptions) (Theme 6), as well as the influence of recent COVID-19 vaccination efforts on potential opportunities for innovative vaccine delivery within communities (e.g., mobile vans, paid leave, and use of social media). Policy-level representatives consistently emphasized the need to engage with school-based clinics to empower adolescents, increase access to vaccination through schools, and educate parents (Theme 7):

*[What is needed is] doing awareness campaigns [in schools], training the youth who are the peer leaders, and what the HPV vaccine is, why they should get it, where they can get it, and also, that there’s an opportunity to get that without parental consent. (LA16, policy)*

Few payers, however, mentioned any direct actions for HPV vaccination. Payers from both regions supported the need to prioritize adolescent immunization but recognized the difficulty to prioritize HPV vaccine specifically because the quality metrics (NCQA, CMS Star ratings, and HEDIS) combine multiple adolescent vaccines (Combo 2). Several payers indicated HPV vaccination was not high on the priority list or of notable interest (Theme 4).

Advocates and policy-level representatives were also aware of current barriers to using immunization registry data to support HPV vaccination improvement strategies within clinics. In some cases, local advocates described supporting clinics by acquiring clinic- and or provider-specific data from the state immunization registry and then filled the resource roles for QI initiatives within clinics, which involved pulling “*HPV rates for a particular*” and then *“putting those rates on a HPV report, show the provider office” (NJ06, advocate).* Policy-level representatives were optimistic for improved implementation of data supported HPV vaccination strategies, but indicated limited motivation in providing support for needed change:

*Providers can also run their own reports to see how many vaccines that they have given to their patients in a certain time period. And we do have some reports that they can sort them by specific vaccines. Those reports are – they’re a little bit more tricky. There’s a few software bugs with them right now. (LA10, policy)*

While there is potential to increase HPV vaccination through external motivators and opportunities (state policies: minor consent, personal belief/religious exemption legislation; payer initiatives: pay for performance for HPV vaccination specifically, vaccine reimbursement in non-primary care settings), these will not be realized without regional political will, advocacy, and implementation.

#### Theme 10: HPV Vaccine Hesitancy and Community Context Varied Across Regions and Result in Varying External Motivators and Opportunities for Change

Community-level differences in HPV vaccine hesitancy across both geographic areas also impacted internal clinic members’ (providers, clinic leaders, and clinic staff) perceived roles in HPV vaccination and communication with parents. Clinic leaders and providers in NJ (NJ22 and NJ28) highlighted HPV vaccine hesitancy among parents more often than those in LA. Advocates in NJ (NJ08) mentioned provider hesitancy to recommend HPV vaccine and the anti-vaccination influence among the parents, while there was limited mention of this form participants in LA. Clinic staff, especially medical assistants, also discussed how they often worked as a cultural/language liaison between the provider and the parent/adolescent and lacked training in HPV vaccine knowledge and communication strategies. Clinic staff in primary care settings indicated that the HPV vaccine has some taboo and hesitancy associated with its purpose, perhaps because HPV is both sexually transmitted and causes cancer (Theme 1):

*I feel like if it might be a little bit taboo, only because of the whole fact that it has to do with like intimacy and stuff like that, which is a very sensitive topic because of the population that we deal with. (LA18, clinic staff)*

Lastly, parents in both states mentioned that schools and their providers (e.g., school-based health centers/nurses) were trusted source of vaccine information and discussed engaging their children in conversations about HPV vaccine/vaccine-related decision-making (Theme 7). Moving forward, effective implementation of HPV vaccination strategies will require an understanding of clinic/parent/adolescent reciprocity, roles, and communication needs in HPV vaccine decision-making (Table 2).

**Table 2:** Practice change model domains and thematic findings in implementation of HPV vaccination strategies within clinic settings

**PCM domains**	**Themes**
Internal clinic motivators and barriers to implementation strategies	
(1) Motivation of key stakeholders & (2) resources for change	Theme 1: conflicting messaging of HPV vaccination complicates strategies • Cancer prevention vaccine messaging conflicts with contexts/settings serving sexual/reproductive health and school-based population • Introducing HPV vaccine at age 9 works for cancer prevention vaccine messaging but interferes with adolescent immunization bundling strategy
	Theme 2: motivation to address missed opportunities but limitations to workflows exist • Standing orders not adopted across clinics due to teaching mission • EHR limitations in identifying missed opportunities or changing workflow for clinic staff
	Theme 3: lack of interoperable EHR and state immunization registry information • Limited bi-directionality between EHR and registry data creates implementation barriers • Concerns around data reliability and completeness
External motivators and barriers for clinic implementation strategies	
(3) Outside motivators & (4) opportunities for change	Theme 4: payers lack motivation for change in HPV vaccination • Current metrics do not target HPV vaccine specifically • Payers showing limited organizational motivation to change incentive structure and acknowledge adolescent immunizations/HPV not at top of priorities
	Theme 5: advocates lead agenda setting at national level and implementation of strategies at local level • Advocates act as liaisons with health departments, work directly with clinics, funding QI initiatives and providing QI resources, leading implementation of strategies at local level • Noted differences in local and regional coalition and policy focus on HPV vaccination
	Theme 6: advocacy, school, and policy presence differs across regions showing need for implementation champions and attention to local context • Policy-level representatives and advocates pushing HPV vaccine awareness at community level • State immunization policies (minor consent in CA; religious exemption in NJ) point to potential strategies beyond school entry mandates for promoting vaccine improvement
	Theme 7: community members (advocates, policy, and parents) indicating opportunity to engage adolescents as vaccine decision-makers • School clinic and leadership noted as important future participants for engagement • Parents indicating support to engage adolescents in vaccine decision-making
Interdependencies across groups and impact on practice change	
(1⇿2) Motivation, innovation, and independence (1⇿3) Motivational reciprocity (2⇿3) Developing change trajectories (3⇿4) External influences on change	Theme 8: motivation for improvement within clinics varies across regions due to available resources for change in regional context • Clinic members are perceptive to payer metrics and shared focus on missed opportunities • Many participants in non-traditional primary care settings serving as HPV champions but perceived unclear/overlapping roles within broader HPV vaccine ecosystem
	Theme 9: lack of specific focus on HPV vaccination from external community members limit motivation among internal clinic members and opportunities for change • Potential to increase HPV vaccination through external motivators such as COVID-19 pandemic but policy and payer groups lack focus/prioritization
	Theme 10: HPV vaccine hesitancy and community context varies across regions resulting in varying external motivators and opportunities for change • More frequent reporting of parental hesitancy by NJ than LA participants • Clinic staff serve as cultural/language liaisons between clinics and communities • Parents highlighting schools and providers as trusted sources of vaccine information and for communication with their children about vaccine decision-making

## Discussion

Advancing the implementation of effective strategies for HPV vaccination in safety-net settings will require adaptation to local clinic and community context. HPV vaccine delivery involves a complex network of participants, internal and external to clinic settings, spanning multiple levels of influence from individual adolescent/parent targets to local and state policy-level representatives. Prior multilevel intervention research to increase HPV vaccine uptake have targeted specific provider and system levels (Niccolai & Hansen, 2015; Smulian et al., 2016; Walling et al., 2016), but rarely include perspectives on EBS implementation and fit from both clinic and community members. Our study highlights the importance and impact of interdependences between multilevel groups on implementation of EBS for HPV vaccine improvement, including engagement between internal clinic members and external advocacy, policy, and payer groups to motivate and provide resources within clinics.

We observe some shared clinic and community contexts in LA and NJ. For example, providers and clinic leaders in both regions demonstrated motivation for improving HPV vaccination, but competing clinic priorities and lack of bi-directional motivation with external members. These shared community contexts, such as lack of local and regional payer (i.e., Medicaid managed care and county health insurance programs) incentives for improving HPV vaccination rates and lack of HPV vaccine prioritization among local policy representatives (i.e., district and state representatives, local county and state health departments) limit the implementation of broader population and policy level strategies and may require more targeted efforts for change. However, we also observe differences in broader community contexts between the two geographic areas, including differing state laws on minor consent for immunizations and availability of state cancer control plans, which can influence state and local agenda setting for HPV vaccine promotion and community-specific factors (e.g., larger Mexican American communities in LA and larger Caribbean and African immigrant communities in NJ) influencing vaccine hesitancy and information channels. Literature is sparse on policy strategies to promote HPV vaccination aside from mandates (Keim-Malpass et al., 2017). Geographically focused improvements in HPV vaccination will require implementation participants to align with and be engaged in the local policy and healthcare environments.

Common and divergent experiences with EBS for HPV vaccine delivery are apparent among clinic and community members. First, clinic members (i.e., providers) across specialty and care delivery settings use divergent messaging about the purpose of HPV vaccine (cancer prevention vs. STI prevention). Community members (i.e., school policymakers and HPV vaccine advocates) perceive the “optional” nature of HPV vaccination differently, which seemed to muddle the motivation and role assignment as to which groups are responsible for improving HPV vaccine uptake. These differing perspectives have the potential to increase misinformation and limit awareness among parents and adolescents as well as increase anticipation of parental hesitancy among providers. Further consistency of HPV vaccine messaging and alignment of messaging within specialty care and non-traditional clinic settings are necessary to reduce internal clinic barriers to vaccination. Second, while clinic members and community members use EHR and state immunization registry data to provide audit and feedback, identify missed opportunities, and/or track/incentize performance, many report structural barriers, such as needing additional staff support and interoperability of systems to fully use data to support EBS. Lastly, while community members indicate potential for higher level strategies, such as payer incentives specific for HPV vaccination or local policy-level representatives’ support for raising HPV vaccine awareness, motivation for and resources to support these potential strategies was limited.

Our findings indicate providers, parents, and advocates recognize adolescents as part of the decision-making process, particularly older adolescents who are in the catch-up age range, or have the opportunity to receive the HPV vaccine through minor consent laws in California. Other studies have also suggested that reliance on or integration of adolescents in decision-making may be greater within immigrant communities, where parental health literacy and access to in-language, culturally appropriate HPV vaccine information may be lower compared to non-immigrant communities (Banas et al., 2017; Gonzalez et al., 2022). To date, adolescent-focused HPV vaccination have been largely led by state and regional HPV vaccine advocates. To achieve long-term sustainability and integration of adolescent-decision making within clinic settings will require broader policy and resource support.

### Limitations

Importantly, we conducted our study over a 14-month period (December 2020 to January 2022) during the COVID-19 pandemic. Thus, we observed the impact of pandemic disruptions to HPV vaccination efforts and preventive care utilization in nearly all interviews. On the one hand, the COVID-19 pandemic created major disruptions in preventive care delivery, resulting in missed vaccine doses among target adolescent age groups (Saxena et al., 2021). Nearly two-thirds of parents in our prior work, among racial/ethnic communities in LA with low HPV vaccine uptake, delayed seeking health care for themselves and their children during the pandemic (Tsui et al., 2022). On the other hand, the pandemic served as an opportunity for innovations in community level vaccine delivery (Omer et al., 2021; Petrova et al., 2021). Other multilevel interventions to improve HPV vaccine uptake during the pandemic also demonstrated effectiveness in other regions and diverse populations (e.g., rural) through community partnerships (Dang et al., 2023; Kepka et al., 2021). Policy-level representatives in LA mentioned greater awareness of immunization strategies within communities and few had worked with clinics directly before the pandemic. It will be important to monitor efforts for HPV vaccination catch-up and how implementation of HPV vaccination strategies will adapt to the changing health care environment and clinic staff shortages and burnout as a result of the pandemic moving forward.

Our study has additional limitations. First, our sampling took place only in two geographic areas (LA and NJ), which was based on the location of the study teams. Both LA and NJ are large metropolitan areas; therefore, our findings may not be readily generalizable to other settings. However, the fact that the two regions are bi-coastal and have diverse racial and ethnic communities and different public health infrastructures for the safety-net settings allowed us to discover underexplored opportunities and participant groups to improve HPV vaccination. Secondly, we aimed to recruit participants based on their involvement with HPV vaccination or adolescent health. While we conducted iterative analyses, with ongoing team discussions for consensus building and purposively sampled for confirming/disconfirming perspectives to achieve thematic saturation, generalizability of study participants was not a primary goal of this qualitative study. Third, every aspect of our study was impacted by disruptions in healthcare delivery due to the COVID-19 pandemic. Ongoing changes in the healthcare system, specifically for immunization and its delivery, likely influenced the participants’ perspectives on implementation strategies to improve HPV vaccinations both within and outside of the clinics. It will be critical to leverage the perspectives captured during this unprecedented time to inform future adaptation of EBS for HPV vaccination and ensure the sustainability of innovations in community-targeted immunization delivery.

Despite these limitations, our findings demonstrate a vital need to apply a more targeted health equity lens within implementation science (Brownson et al., 2021; Chinman et al., 2017). Observed regional differences in relationships and perspectives across clinic and community members also point to the need for adaptation of strategies to fit the local context and populations. While prior HPV vaccination studies have utilized implementation frameworks to examine internal clinic processes, few have considered the dynamic interdependencies among providers, clinics, health systems, and the regional community and policy contexts (Anderson et al., 2005; Miller et al., 2001; Stroebel et al., 2005). Prior work conducting an environmental scan and community feedback indicated HPV vaccination inequities can be greatly reduced in diverse regions, such as LA County, if a multilevel, multicultural, and multilingual approach is taken (Baezconde-Garbanati et al., 2017).

## Conclusions

Our findings inform the field of implementation science for preventive care delivery by demonstrating the importance of interdependencies within regional clinic and community contexts that impact population-level implementation efforts. Scaling up and spreading of EBS for HPV vaccination implementation to address disparities in cervical and other HPV-associated cancers will require the following: 1) expanding local workforce roles and scope of practice for adolescent health, both within clinics (HPV vaccine education for clinic staff) and outside of clinics (policy to expand HPV vaccine access in reproductive health/school-based settings); 2) strengthening the bi-directional motivation to adopt effective strategies for HPV vaccination between internal clinic members (e.g., workflow to address missed opportunities) and external community members (e.g., quality metrics specific to HPV vaccination); and 3) responsiveness to the local context of structural inequities and social determinants that lead to increased HPV vaccination hesitancy and other barriers among marginalized communities.
